# Clinical and microbiological characteristics of and risk factors for bloodstream infections among patients with extracorporeal membrane oxygenation: a single-center retrospective cohort study

**DOI:** 10.1038/s41598-022-19405-z

**Published:** 2022-09-05

**Authors:** Eun Hwa Lee, Ki Hyun Lee, Se Ju Lee, Jinnam Kim, Yae Jee Baek, Jin Young Ahn, Su Jin Jeong, Nam Su Ku, Jun Yong Choi, Joon-Sup Yeom, Young Goo Song, Jung Ho Kim

**Affiliations:** Division of Infectious Diseases, Department of Internal Medicine and AIDS Research Institute, Yonsei University College of Medicine, Yonsei University Health System, Seoul, South Korea 50-1, Yonsei-ro, Seodaemun-gu, 03722

**Keywords:** Microbiology, Diseases, Risk factors

## Abstract

Extracorporeal membrane oxygenation (ECMO) provides hemodynamic and oxygenation support to critically ill patients. Due to multiple catheter cannulations, patients on ECMO are vulnerable to bloodstream infections (BSIs). We aimed to investigate the incidence, clinical characteristics, risk factors, and microorganisms associated with BSIs during ECMO. This single-center retrospective cohort study was conducted between January 2015 and May 2021. Patients aged 18 years or older with an ECMO duration of > 48 h for cardiogenic or respiratory support were included in the study. Patients who developed bacteremia or candidemia from 12 h after ECMO cannulation to 7 days after de-cannulation were included. The clinical factors between non-BSI and BSI were compared, along with an analysis of the risk factors associated with BSI during ECMO. A total of 480 patients underwent ECMO for cardiogenic shock (n = 267, 55.6%) or respiratory failure (n = 213, 44.4%) during the study period. The incidence was 20.0 episodes per 1000 ECMO-days. Approximately 20.2% (97/480) and 5.4% (26/480) of the patients developed bacteremia and candidemia, respectively. The median numbers of days of BSI development were 8.00 days for bacteremia and 11.0 days for candidemia. The most common pathogens were methicillin-resistant coagulase-negative staphylococci (n = 24), followed by vancomycin-resistant *Enterococcus* (n = 21). Multivariable logistic analysis demonstrated that hemodialysis (odds ratio [OR] 2.647, p < 0.001), veno-arterial-venous mode (OR 1.911, p = 0.030), and total ECMO duration (OR 1.030, p = 0.007) were significant risk factors for bacteremia. The total ECMO duration was the only risk factor associated with candidemia (OR 1.035, p = 0.010). The mortality rate was significantly higher in the bacteremia (57.7%) and candidemia (69.2%) groups than that in the non-BSI group (43.6%). BSI is a common complication of patients receiving ECMO support and is associated with poor clinical outcomes. Determining the type of frequently isolated organisms and the median onset time of BSI would help in the selection of appropriate prophylactic antibiotics or antifungal agents.

## Introduction

Extracorporeal membrane oxygenation (ECMO) provides cardiogenic and respiratory support for emergency resuscitation, trauma, cardiogenic shock, and respiratory failure patients^1^. During ECMO, a large volume of blood is drained from the patient, passes through the circuits, is oxygenated via a membrane oxygenator, and returns the blood to the patient^2^. Depending on the ECMO mode, ECMO catheters are inserted into either the arterial or venous circulation. The veno-venous ECMO provides respiratory assistance, while the veno-arterial ECMO provides cardiac and respiratory support^1,3^. Since ECMO requires multiple cannulations in major vessels, patients receiving ECMO support are vulnerable to bloodstream infections (BSIs). Moreover, additional arterial lines, multiple central lines, and dialysis catheters for renal replacement therapy are often required^4^. Unfortunately, even if BSI occurs during ECMO, ECMO catheter removal is not often feasible as the patients are hemodynamically unstable^5^. In previous studies, the prevalence of BSI was 3–18%, with the incidence ranging from 2.98 to 20.55 episodes per 1000 ECMO days^2^. BSIs accounted for the majority of infections (69.1%) occurring during ECMO, followed by surgical site infections (10.9%) and respiratory infections (7.3%)^6^. Although previous studies presented the risk factors and clinical characteristics of various types of nosocomial infections occurring during ECMO, this study analyzed BSI in ECMO patients in terms of incidence, clinical features, risk factors, common causal microorganisms, and their antibiotic susceptibility. This study also evaluated BSI during ECMO from a hospital-associated infection perspective and identified a patient group that might benefit from prophylactic antibiotic treatment and blood culture surveillance.

## Methods

### Patient selection

In this single-center, retrospective, observational cohort study, adult patients who underwent ECMO at Severance Hospital between January 2015 and May 2021 were initially screened (n = 769). Severance Hospital is a 2400-bed tertiary referral hospital that performs various lung and heart surgical procedures, including organ transplantations. Patients who met the following criteria were included in the study: (1) aged 18 years or older, (2) who received ECMO support beyond 48 h, and (3) who received ECMO for the first time. Patients who received repeated ECMO were excluded from the analysis. This study was approved by the institutional review board of Severance Hospital (No. 4-2021-1587). The requirement for informed consent was waived owing to the retrospective nature of the study. All procedures and treatments observed current guidelines and regulations.

### Data collection

Patients’ clinical information, demographics, and laboratory data at the initiation of ECMO were obtained from the Severance Clinical Research Analysis Portal. The data included age, sex, body mass index, underlying medical conditions, and medications. Detailed information related to ECMO was obtained from a daily ECMO report written by physicians. This information included the reasons for ECMO support (cardiogenic vs. respiratory failure), mode of ECMO (veno-arterial [VA], veno-venous [VV], or veno-arterial-venous [VAV]), cannulation site of ECMO catheters, location of catheter cannulation (intensive care unit, operating room, emergency room, or intervention room), type of ECMO machine (prolonged life support [PLS] or emergency bypass system [EBS]), and total time of ECMO implantation. Additionally, data on the application of renal replacement therapy were also collected. Data on antibiotic usage, blood culture results during ECMO support, type of organisms isolated, and antibiotic sensitivity test results were collected. The antibiotics administered prior to or during ECMO cannulation were analyzed in this study.

### ECMO procedure

Intensive care specialists, cardiologists, or cardiothoracic surgeons determined the need for ECMO initiation by evaluating the patient’s medical condition. The ECMO techniques used in our institution have been described in a previously published study^7^. Cannulation was performed using the Seldinger technique, and the preferred cannulation sites were the femoral and internal jugular veins for VV ECMO and the femoral vein and artery for VA ECMO. If additional cardiogenic support was required for patients receiving VV ECMO, an additional arterial catheter was inserted and the mode was switched to VAV ECMO. For the analysis, the specific mode with the highest number of catheters inserted was determined for each ECMO mode. All patients who underwent ECMO cannulation were infused with heparin (2000 IU). The target activated coagulation time was 180–200 s. The Capiox EBS (Terumo Co., Ltd., Tokyo, Japan) or Bioline heparin-coated Quadrox PLS circuit system (Maquet Cardiopulmonary, Hirrlingen, Germany) was used in all patients. All ECMO patients requiring addition of continuous renal replacement therapy (CRRT) used separate circuits for each system.

### Definition of bacteremia during ECMO support

All blood culture results of patients were evaluated, and patients who developed bacteremia 12 h after ECMO cannulation until 7 days after de-cannulation were identified. If the blood sample obtained less than 12 h after ECMO cannulation was positive for bacterial or fungal growth, bacteremia or candidemia was assumed to have existed prior to ECMO and excluded from the BSI group. The detection of coagulase-negative *Staphylococcus* (CoNS) or any common commensal organisms defined by the Center for Disease Control in one blood culture without compatible clinical signs or other supporting evidence of infection was considered contamination^8^. Multidrug-resistant organisms (MDROs) are defined as organisms that are non-susceptible to at least one agent in three or more antimicrobial categories^9^.

### Clinical outcomes

Patients were followed up until discharge or death. The primary endpoint was the development of bacteremia or candidemia during ECMO. In-hospital mortality data were collected from the electronic medical records.

### Statistical analysis

Statistical analysis was performed using Statistical Package for Social Sciences version 26 (SPSS, Chicago, IL, USA). Continuous variables were expressed as means and standard deviations or medians with interquartile ranges, whereas categorical variables were expressed as numbers and percentages. The Mann–Whitney U test was used to compare continuous variables, while the chi-square test and Fisher’s exact test were used to compare categorical variables. The survival curve was expressed using a Kaplan–Meier curve, and the p values used in comparing non-BSI with bacteremia or candidemia were obtained using a log-rank test. Univariable and multivariable logistic regression analyses were used to compare the factors associated with BSI in patients receiving ECMO support. The odds ratios (ORs) and their corresponding 95% confidence intervals (CIs) were calculated. All tests were two sided, and a p value of less than 0.05 were considered significant.

### Ethics approval and consent to participate

This study was approved by the institutional review board of Severance Hospital (No. 4-2021-1587). The requirement for informed consent was waived owing to the retrospective design of the study.

## Results

### Baseline characteristics

**Figure 1:**
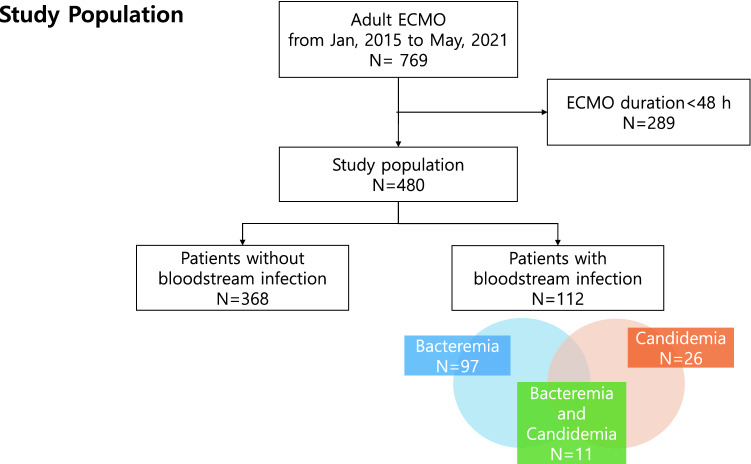
Study flow chart. *ECMO* Extracorporeal membrane oxygenation.

A total of 769 adult patients received ECMO for cardiogenic shock or respiratory failure between January 2015 and May 2021. Of them, 289 patients were excluded from the analysis. A total of 480 patients were included in the final analysis, of whom 368 and 112 comprised the non-BSI and BSI groups, respectively. The total duration of ECMO use was 5604.8 ECMO-days, and the BSI incidence was 20.0 episodes per 1000 ECMO-days. Approximately 20.2% (97/480) and 5.4% (26/480) of the patients developed bacteremia and candidemia, respectively; meanwhile, both bacteremia and candidemia occurred sequentially or simultaneously in 2.3% (11/480) of the patients (Fig. 1).

Table 1 displays the baseline characteristics of patients in the non-BSI and BSI groups (bacteremia and candidemia group). The mean age of the participants was 56.3 ± 14.5 years, and 65.4% of them were men. Patients in the BSI group were older than those in the non-BSI group (58.8 vs. 55.5, p = 0.024). The BSI group had a lower proportion of patients with congestive heart failure (CHF) (42.9% vs. 53.5% vs. p = 0.048); moreover, the candidemia group had a lower proportion of patients with CHF than in the non-BSI group (30.8% vs. 53.5% p = 0.026). The number of patients requiring hemodialysis, including intermittent or continuous, was higher in the BSI group (59.8% vs. 41.1%, p = 0.001), especially in the bacteremia group. The median length of pre-ECMO hospital stay was significantly longer in the bacteremia (23.0 days) and candidemia (25.5 days) groups than in the non-BSI group (13.0 days). Over 70% of the patients were already receiving antibiotics, and more than 60% were using antibiotics with *Pseudomonas* coverage. The rate of antibiotic use before ECMO did not differ between the non-BSI and BSI groups.

**Table 1:** Comparison between the bacteremia and candidemia groups.

	All patients	Patients without	Patients with BSI§		*p value	†p value
		Bloodstream infection (BSI)	Bacteremia	Candidemia		
**Baseline characteristic**						
Number of patients	480	368	97	26		
Age, years	56.3 ± 14.5	55.5 ± 14.9	58.9 ± 13.3	58.5 ± 12.7	**0.031**	0.323
Male	314 (65.4)	242 (65.8)	63 (64.9)	14 (53.8)	0.881	0.215
BMI, kg/m^2^	1.72 ± 0.23	1.71 ± 0.23	1.73 ± 0.23	1.73 ± 0.19	0.442	0.675
**Underlying conditions**						
Diabetes mellitus	219 (45.6)	164 (44.6)	49 (50.5)	14 (53.8)	0.295	0.352
Congestive heart failure	245 (51.0)	197 (53.5)	43 (44.3)	8 (30.8)	0.107	**0.026**
COPD	91 (19.0)	71 (19.3)	18 (18.6)	2 (7.7)	0.870	0.193
Chronic liver disease	22 (4.6)	19 (5.2)	2 (2.1)	1 (3.8)	0.273	> 0.999
Hemodialysis	218 (45.5)	151 (41.1)	58 (59.8)	15 (57.7)	**0.001**	0.104
Solid organ transplantation	173 (36.0)	130 (35.3)	39 (40.2)	10 (38.5)	0.374	0.739
Connective tissue disease	16 (3.3)	11 (3.0)	4 (4.1)	2 (7.7)	0.528	0.208
On immunosuppressant therapy	288 (60.0)	214 (58.2)	63 (64.9)	18 (69.2)	0.134	0.272
Charlson Comorbidity Index score	4.40 ± 2.34	4.32 ± 2.42	4.62 ± 2.00	4.46 ± 2.08	0.269	0.775
Pre-ECMO hospital stay days	16.0 [7.0–36.0]	13.0 [7.0–34.0]	23.0 [12.5–45.0]	25.5 [16.8–46.3]	**0.025**	**0.040**
**Reason for ECMO**						
Cardiogenic	267 (55.6)	215 (58.4)	45 (46.4)	10 (38.5)	**0.034**	**0.049**
Respiratory	213 (44.4)	153 (41.6)	52 (53.6)	16 (61.5)		
**Mode of ECMO**						
VA	244 (50.8)	202 (54.9)	36 (37.1)	8 (30.8)	**0.004**	**0.036**
VV	130 (27.1)	97 (26.4)	26 (26.8)	10 (38.5)	0.993	0.160
VAV	106 (22.1)	69 (18.8)	35 (36.1)	7 (26.9)	**0.001**	0.301
**ECMO system**^**‖**^						
PLS	254 (55.2)	182 (52.1)	63	17	0.077	0.229
EBS	206 (44.8)	167 (47.9)	33	8		
**Antibiotics usage**						
Any antibiotics used prior to ECMO	357 (75.0)	267 (73.4)	77 (79.4)	22 (84.6)	0.225	0.208
*Pseudomonas* coverage^‡^	299 (83.8)	221 (82.8)	67 (87.0)	19 (86.4)	0.374	0.777
Glycopeptides	219 (61.3)	159 (59.6)	52 (67.5)	15 (68.2)	0.067	0.147
Anti-fungal agents	49 (13.7)	33 (12.4)	15 (19.4)	2 (7.7)	0.061	> 0.999
Total duration of ECMO (days)	7.18 [4.17–12.71]	6.11 [3.78–10.82]	13.0 [7.09–17.18]	17.2 [7.51–27.17]	**0.003**	**0.001**
In-hospital mortality	227 (47.4)	160 (43.6)	56 (57.7)	18 (69.2)	**0.013**	**0.012**

Data are expressed as number(percent), average ± standard deviation, or median [25% and 75%].

p values with statistical significance is shown in bold text.

*BMI* body-mass index, *COPD* chronic obstructive pulmonary disease, *ECMO* extracorporeal membrane oxygenation, *VA* veno-arterial, *VV* veno-venous, *VAV* veno-arterial-venous, *PLS* prolonged life support, *EBS* emergency bypass system, *BSI* bloodstream infection.

*p value is used to compare patients without BSI and patients with bacteremia.

^†^p value is used to compare patients without BSI and patients with candidemia.

^‡^Antibiotics with *Pseudomonas* coverage: piperacillin/tazobactam, cefepime, ceftazidime, imipenem, meropenem, ciprofloxacin, levofloxacin, and aztreonam.

^§^11 patients developed both bacteremia and candidemia during ECMO support.

^‖^ECMO system unknown (n = 20).

### Association of BSI with the reason for ECMO and mode of ECMO

Of the 480 patients, 267 received ECMO for cardiogenic support, and 213 received ECMO for respiratory support. The BSI group had a higher proportion of patients who received ECMO for respiratory support (53.6% vs. 41.6%, p = 0.027) compared with that of the non-BSI group, indicating that patients requiring ECMO for respiratory support had a higher risk of developing BSI (Tables 1, 2). The VA, VV, and VAV ECMO modes were used in 244, 130, and 106 patients, respectively. The frequent reasons for receiving VA support were sudden cardiac arrest, acute myocardial infarction, acute decompensated heart failure, and refractory arrhythmia. For VV support, bridge-to-lung transplantation and acute respiratory distress syndrome were the most common clinical conditions that require ECMO. When the mode of ECMO support was compared, higher portion of patients in VAV mode developed BSI. The rate of BSI in VAV mode was 34.9% (37/106 cases), VA was 25.4% (33/130 cases), and VV was 17.2% (42/244 cases).

**Table 2:** Clinical variables associated with bloodstream infection (bacteremia, candidemia).

	Bacteremia				Candidemia			
	Univariable*		Multivariable*		Univariable†		Multivariable*	
	OR (95% CI)	p value	OR (95% CI)	p value	OR (95% CI)	p value	OR (95% CI)	p value
**Baseline characteristic**								
*Age*	*1.017* *(1.001–1.033)*	*0.043*	1.018 (0.999–1.036)	0.058	1.014 (0.986–1.044)	0.323		
Male	1.037 (0.648–1.657)	0.881			1.653 (0.742–3.681)	0.218		
BMI	1.463 (0.556–3.845)	0.441			1.458 (0.657–3.239)	0.354		
**Underlying conditions**								
Diabetes mellitus	1.270 (0.811–1.987)	0.296			1.458 (0.657–3.239)	0.354		
Congestive heart failure	0.691 (0.441–1.084)	0.108			*0.388* *(0.165–0.915)*	*0.031*	0.425 (0.177–1.016)	0.054
COPD	0.953 (0.537–1.692)	0.870			0.350 (0.081–1.514)	0.160		
Chronic liver disease	0.387 (0.089–1.690)	0.207			0.737 (0.095–5.732)	0.770		
Hemodialysis	*2.217* *(1.348–3.357)*	*0.001*	*2.647* *(1.547–4.528)*	< *0.001*	1.960 (0.876–4.384)	0.102		
Solid organ transplantation	1.231 (0.778–1.948)	0.374			1.149 (0.507–2.605)	0.739		
Connective tissue disease	1.396 (0.435–4.484)	0.575			2.712 (0.569–12.936)	0.211		
On immunosuppressant therapy	1.333 (0.837–2.124)	0.226			1.612 (0.683–3.801)	0.276		
Charlson comorbidity Index score	1.055 (0.960–1.159)	0.269			1.024 (0.869–1.207)	0.774		
**Antibiotics usage prior to ECMO**								
Any antibiotics used prior to ECMO	1.399 (0.812–2.410)	0.227			1.991 (0.669–5.923)	0.216		
*Pseudomonas* coverage	1.395 (0.668–2.913)	0.376			1.347 (0.383–4.737)	0.643		
Glycopeptides	1.519 (0.969–2.380)	0.068			1.801 (0.805–4.028)	0.152		
Anti-fungal	1.857 (0.963–3.580)	0.065			0.848 (0.192–3.750)	0.828		
**Reason for ECMO**								
Cardiogenic	Reference				Reference			
*Respiratory*	*1*.*624* *(1.036–2.546)*	*0.035*	0.910 (0.481–1.723)	0.773	2.223 (0.982–5.032)	0.055		
**Type of mode**								
VA	Reference				Reference			
VV	1.504 (0.860–2.632)	0.153			1.806 (0.784–4.156)	0.165		
VAV	*2.846* *(1.659–4.882)*	< *0.001*	*1.911* *(1.065–3.429)*	*0.030*	1.612 (0.648–4.012)	0.305		
**ECMO system**								
EBS	Reference				Reference			
PLS	*1.700* *(1.066–2.712)*	*0.026*	1.656 (0.862–3.182)	0.130	1.695 (0.712–4.039)	0.233		
**Location of cannulation**								
Intensive care unit	Reference				Reference			
Emergency room	0.316 (0.070–1.418)	0.132			No case			
Intervention room	*0.395* *(0.190–0.822)*	*0.013*	0.955 (0.408–2.233)	0.915	0.397 (0.092–1.723)	0.217		
Operating room	0.529 (0.242–1.155)	0.110			1.378 (0.453–4.190)	0.572		
Pre-ECMO hospital stay days	*1.006* *(1.001–1.012)*	*0.015*	0.997 (0.989–1.005)	0.443	1.007 (1.000–1.014)	0.057		
*Total* *duration* *of* *ECMO*	*1.039* *(1.017–1.061)*	< *0.001*	*1.030* *(1.008–1.053)*	*0.007*	*1.036* *(1.010–1.063)*	*0.006*	*1.035* *(1.008–1.061)*	*0.010*

p values with statistical significance is shown in italics text.

*Indicates bacteremia group.

^†^Indicates candidemia group.

*BMI* body-mass index, *COPD* chronic obstructive pulmonary disease, *ECMO* extracorporeal membrane oxygenation, *VA* veno-arterial, *VV* veno-venous, *VAV* veno-arterial-venous, *PLS* prolonged life support, *EBS* emergency bypass system.

### Duration of ECMO and risk of BSI

**Figure 2:**
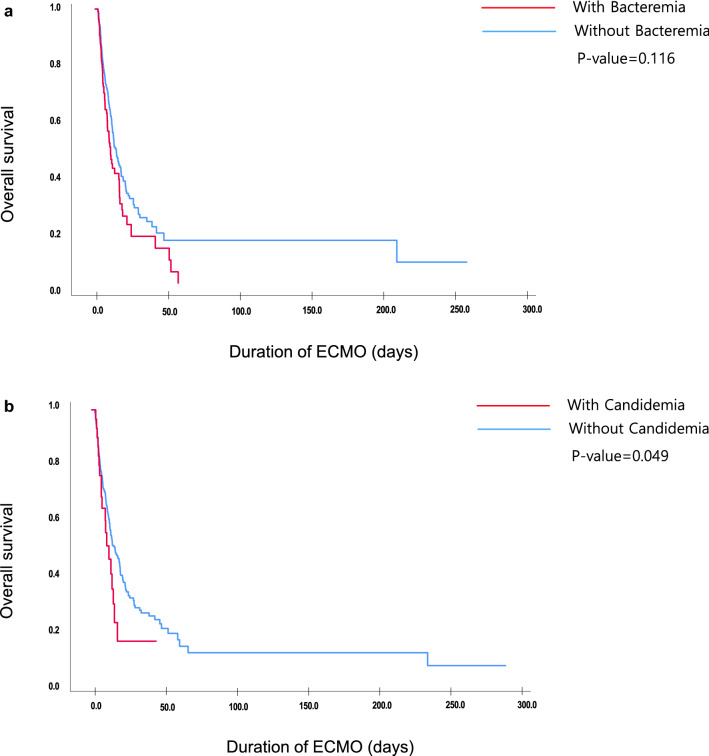
(**a**) Kaplan–Meier curve for non-BSI vs. Bacteremia group. *BSI* bloodstream infection, *ECMO* extracorporeal membrane oxygenation. (**b**) Kaplan–Meier curve for non-BSI vs. Candidemia group. *BSI* bloodstream infection, *ECMO* extracorporeal membrane oxygenation.

The total duration of ECMO support in median days was higher in the bacteremia and candidemia groups (13.0 and 17.2 days) compared with that in the non-BSI group (6.11 days) (p = 0.003 and 0.001, respectively). The in-hospital mortality rate was also higher in the bacteremia and candidemia groups (57.7% and 69.2%, respectively) compared with that in the non-BSI group (43.6%) (p = 0.013 and 0.012, respectively). The Kaplan–Meier curves in Fig. 2a,b show that the mortality curve rapidly dropped in both the bacteremia and candidemia groups compared with that in the non-BSI group.

**Figure 3:**
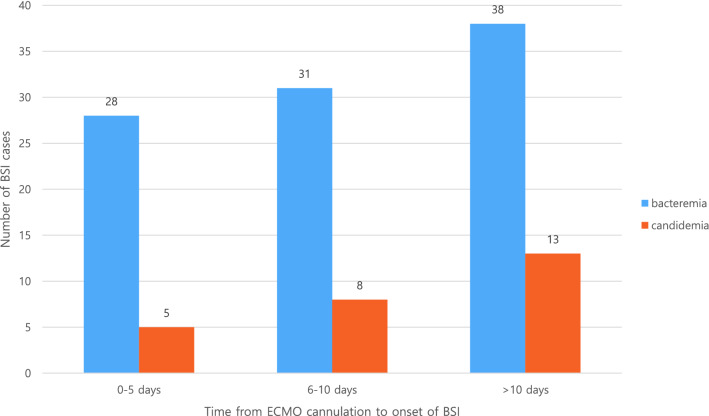
The number of BSI cases at different periods after ECMO cannulation. *BSI* bloodstream infection, *ECMO* extracorporeal membrane oxygenation.

Figure 3 shows the number of BSI cases at different periods after ECMO cannulation. The number of BSI cases increased as ECMO cannulation time increased. Approximately 71.1% of patients developed BSI after more than 6 days of ECMO cannulation. The median onset time of BSI was 8.0 days (range 5.0–16.0 8.0): 8.0 (5.0–13.5) for bacteremia and 11.0 (6.0–20.0) for candidemia, respectively. According to ECMO mode, the median numbers of days of BSI onset was 7.0 (6.0–20.0) in patients who used the VA mode, 9.0 days (2.5–20.0) in patients who used the VV mode, and 13.5 median days (8.75–22.5) in patients who used the VAV mode.

### Risk factor associated with BSI in ECMO

The risk factors associated with bacteremia and candidemia during ECMO were analyzed using univariable and multivariable logistic regression analyses (Table 2). In univariable analysis, age, hemodialysis, respiratory support, VAV mode, ECMO system with PLS, cannulation procedure performed in the intervention room, pre-ECMO hospital days, and total duration of ECMO were the risk factors of bacteremia. In the multivariable analysis, only hemodialysis (OR 2.647, 95% CI 1.547–4.528, p < 0.001), VAV mode (OR 1.911, 95% CI 1.065–3.429, p = 0.030), and total duration of ECMO (OR 1.030, 95% CI 1.008–1.053, p = 0.007) were the significant clinical factors associated with an increased risk of bacteremia. Moreover, only the total duration of ECMO (OR 1.035, 95% CI 1.008–1.061, p = 0.010) was associated with the risk of candidemia.

### Major causal pathogens of BSI during ECMO

Table 3 lists the frequently isolated causal pathogens of bacteremia or candidemia during ECMO along with the first isolated time of each organism (in median days). Among the gram-negative organisms, *Acinetobacter* *baumannii* and *Klebsiella* *pneumoniae* were the most commonly detected pathogens of BSI during ECMO. Both organisms displayed multidrug resistance to antibiotics. All isolated *Escherichia* *coli* was either extended-spectrum beta-lactamase-producing (5 out of 6) or multidrug-resistant organism (1 out of 6). Coagulase-negative staphylococcus (methicillin resistant) and enterococcus (vancomycin resistant) were common pathogens in gram-positive organisms. *Candida* *tropicalis* was the most common Candida species, followed by *Candida* *parasilosis* and *Candida* *albicans*.

**Table 3:** Type of organism isolated from blood culture.

Type of organism	Number of cases	First isolated time in days (median, range)
**Gram negative**		
*Acinetobacter* *baumannii*	18	8.1 (4.56–15.3)
*Enterobacter* *cloacae*	1	4.9
*Escherichia* *coli*	6	5.8 (1.2–12.9)
*Klebsiella* *pneumoniae*	18	8.4 (5.6–14.2)
*Morganella* *morganii*	1	29.0
*Orchrobactrum* sp.	1	0.2
*Pseudomonas* *aeruginosa*	3	8.3 (6.2–17.1)
*Serratia* *marcescens*	1	16.4
*Stenotrophomonas* *maltophilia*	5	7.3 (6.4–9.2)
**Gram positive**		
Coagulase negative staphylococcus (methicillin-resistant)	24	7.9 (5.0–12.2)
Coagulase negative staphylococcus (methicillin-sensitive)	4	8.1 (5.7–10.4)
*S.* *aureus* (methicillin-resistant)	4	4.1 (1.5–17.6)
*S.* *aureus* (methicillin-sensitive)	5	2.3 (0.3–19.2)
*Corynebacterium* sp.	2	14.3 (13.3–15.3)
*Enterococcus* (vancomycin-resistant)	21	16.3 (6.4–20.7)
*Enterococcus* (vancomycin-sensitive)	11	9.3 (6.9–11.8)
**Candida**		
*Candida* *albicnas*	8	7.6 (4.2–9.8)
*Candida* *glabrata*	1	19.2
*Candida* *parasilosis*	8	28.25(20.2–39.9)
*Candida* *tropicalis*	11	10.0 (5.5–16.3)

Data are expressed as number or median with range (25–75%).

**Figure 4:**
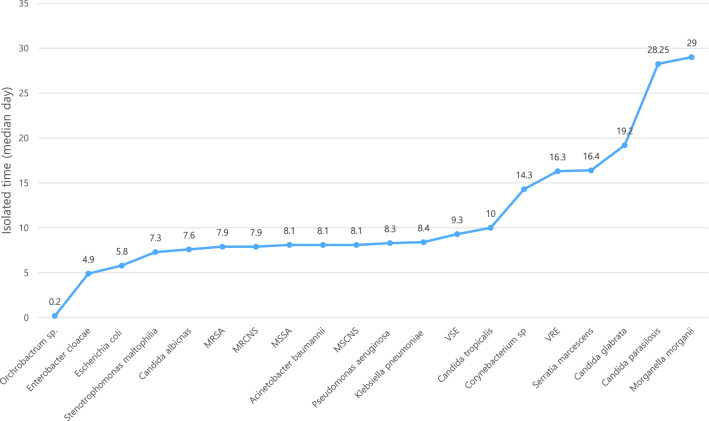
Type of organisms isolated in chronologic order. *MRSA* Methicillin-resistant *Staphylococcus* *aureus*, *MRCNS* Methicillin-resistance coagulase negative *Staphylococci*, *MSSA* Methicillin-sensitive coagulase negative *Staphylococci*, *VSE* Vancomycin sensitive *Enterococci*, *VRE* Vancomycin resistant *Enterococci.*

Figure 4 shows the microorganisms isolated on the first median days, in chronological order, to visualize the pathogens responsible for developing BSI in the early or later phase of ECMO. Bacteremia due to *Enterobacter* *cloacae* and *Escherichia* *coli* occurs in the earlier phase (less than 7 days) after ECMO cannulation. By contrast, vancomycin-resistant enterococcus, *Candida* *glabrata*, *Candida* *parasilosis*, and *Morganella* *morganii* were causal pathogens of BSI during ECMO, which developed in the later phase (after 15 days) of ECMO cannulation.

Table 4 shows the cases in which the causal pathogens were isolated from other sources before causing BSI. Furthermore, the causative microorganisms in BSI 37 out of 98 (37.8%) patients in the bacteremia group and 9 out of 26 (34.6%) patients in the candidemia group were previously isolated from other sources, such as sputum, urine, bronchial alveolar lavage, pleural drainage catheter, swab culture, endotracheal tube, or stool. Approximately 25.5% and 23.1% of bacteremia and candidemia pathogens, respectively, were isolated after ECMO cannulation. The most commonly isolated sources were sputum and bronchial alveolar lavage for bacteremia and sputum and urine for candidemia.

**Table 4:** Causal pathogen isolated in other sources before bacteremia or candidemia.

	Bacteremia	Candidemia
No. of total BSI cases	98	26
Causal pathogen isolated in other sources	37 (37.8)	9 (34.6)
Causal pathogen isolated in other sources after ECMO cannulation	25 (25.5)	6 (23.1)
**Site of isolation**		
Sputum	16 (43.2)	3 (33.3)
Blood	1 (2.7)	0
Urine	5 (13.5)	4 (44.4)
Bronchial alveolar lavage	11 (29.7)	1 (11.1)
Endotracheal tube	7 (18.9)	1 (11.1)
Stool	1 (2.7)	0

Data are expressed as number (percent).

*BSI* bloodstream infection, *ECMO* extracorporeal membrane oxygenation.

## Discussion

BSI is a critical complication of ECMO since BSI during ECMO is associated with increased mortality and morbidity^2^. The BSI rate during ECMO was higher in adults than in neonates and children^10^. Therefore, preventing the occurrence of BSI in adults is crucial. However, there are insufficient data to guide the active surveillance or the selection of prophylactic antibiotics.

The incidence of BSI during ECMO (23.3%) reported in this study was higher than the previously reported incidence of 3–18%^2,11,12^. This high incidence might be attributed to our extended definition of BSI during ECMO, which included BSI occurring up until 7 days after de-cannulation. Since the skin barrier is broken during ECMO cannulation and requires sufficient time to heal depending on the patient’s medical condition after catheter removal, the incidence of BSI was evaluated for an extended period.

The significant risk factors associated with bacteremia during ECMO were hemodialysis, VAV mode, and total duration of ECMO. The hemodialysis and VAV modes may be related to the need for additional indwelling catheters. Many ECMO patients require renal replacement therapy during ECMO for acute kidney injury (AKI) due to hemodynamic instability. To provide hemodialysis (HD) or CRRT, a permanent catheter or a temporary catheter is required. In this study, we did not determine whether the risk of BSI differs in patients requiring hemodialysis due to end-stage renal disease (ESRD) or in those requiring hemodialysis due to AKI. A previous study reported a risk of BSI in patients with elevated creatinine levels^13^. Therefore, impaired renal function requiring hemodialysis is associated with a risk of BSI during ECMO.

The critical risk factors associated with BSI development were extended hospital stay and total duration of ECMO. Multiple studies demonstrated that the total duration of ECMO was the most critical risk factor of BSI^2,13–15^. The longer the patient stays in the ward or intensive care unit, the higher the risk of acquiring nosocomial pathogens. Therefore, shortening the total ECMO duration and hospital stay should be considered if the patient’s hemodynamic stability is restored.

Different ECMO modes were also associated with BSI during ECMO. The reason for ECMO support was used to determine the initial mode of ECMO. In Sun et al.’s study, the risk for using the VV mode was relatively higher (OR 4.473, CI 1.001–19.977, p = 0.050), probably because patients requiring respiratory support may have already developed infections prior to receiving ECMO support^6^. Other studies have also reported that most BSI cases were due to the use of respiratory ECMO^5,16^. VV ECMO was associated with BSI due to gram-negative bacteremia compared with the VA mode^16^. In this study, multivariable logistic analysis proved that VAV mode was associated with increased risk of developing bacteremia (OR 2.846, p < 0.001). Although not significant, the VV mode was also associated with an increased risk of developing bacteremia (OR 1.504, p = 0.153). As the VV mode is initially applied to patients with respiratory failure due to pneumonia, acute respiratory distress syndrome, or lung transplantation, such patients may have a higher risk of carrying the associated microorganisms in the lungs.

As the circuit regulates the body temperature of ECMO patients, clinicians often face difficulties in detecting the signs and symptoms of infection^17^. Leukocytosis and C-reactive protein levels are useful laboratory markers for evaluating the risk of infection in the absence of fever. In particular, if patients on ECMO support for longer than 10 days present with leukocytosis and low-grade fever, infection should be suspected^4^. Our study results showed that the median onset time of BSI was 8.0 days for bacteremia and 11.0 days for candidemia. Therefore, patients who demonstrate signs of infection beyond 8 or 11 days after ECMO cannulation, with elevated levels of laboratory markers, could be considered for prophylactic antibiotic or antifungal treatment due to the possibility of infection, especially BSI. Taking the median onset time of BSI into account for each ECMO system, patients using VA, VV, and VAV ECMO longer than 7, 9, and 13.5 days, respectively, should be carefully assess the signs of infection.

Consistent with the findings of previous studies, our study also noted CoNS as the most frequently isolated microorganism in BSI during ECMO^14^. Membrane oxygenators have large artificial contact surfaces with blood and are a potential source of infection during ECMO^2^. CoNS can bind to fibronectin, colonize membrane surfaces, and produce biofilms^18^. However, routine use of glycopeptides might not be helpful in ECMO patients. Clinicians often add glycopeptides when infections are suspected in patients on ECMO support. However, inappropriate antibiotic use increases the risk of acquiring multidrug resistance. According to our data, both the non-BSI and BSI groups used antibiotics including antipseudomonal agents and glycopeptides. However, the use of these antibiotics did not reduce the incidence of BSI. Approximately 42% of ECMO centers systematically administer antibiotics to all patients, with varying timings^19^. The administration of antibiotics without consideration of the possibility of infection has no benefits. Because most nosocomial infections develop after a median of 9.5 days, our data do not support the need for early antimicrobial prophylactic treatment, which may target drug-resistant pathogens. In other studies, prior exposure to carbapenem was associated with *Candida* BSI^16^.

Fungal prophylaxis may be helpful because *Candida* BSI is associated with higher mortality in ECMO patients than in the general population^19^. A previous study demonstrated that appropriate fungal prophylaxis in pediatric patients decreased the incidence of fungal infections^17^. Since the median onset time for candidemia is approximately 11 days after ECMO cannulation, an anti-fungal agent may be considered for patients who show signs of infection 11 days after ECMO insertion^2,20^.

Finally, this study emphasizes the importance of environmental control in hospitals. According to our data, most causal microorganisms were isolated from the respiratory samples. Previous studies have reported that the prevalence of hospital-acquired infection during ECMO is 10–12%, and multidrug-resistant pathogens were detected in 28.6% of patients^17,21^. With extended hospital stay, the risk of acquiring various microorganisms, including MDROs, is higher. In this study, several microorganisms isolated from blood cultures were subjected to MDROs. Knowing the commonly isolated microorganisms of the intensive care unit of the institution and the colonizing organism is essential for selecting the appropriate empirical antibiotics for treating BSI in ECMO patients.

Our study has some limitations. It was conducted in a single-center and was retrospective and observational in nature. Information on additional devices, such as intra-aortic balloon pumps, the use of distal perfusion catheter insertion, and a temporary pacemaker, may help evaluate the impact of BSI on ECMO. Complications such as AKI, distal perfusion failure, and skin necrosis might be other risk factors associated with BSI during ECMO support.

## Conclusions

BSI is a common complication of ECMO and is associated with poor clinical outcomes. The risk factors associated with BSI in ECMO should be well established to improve the prognosis. Considering the type of frequently isolated organisms and the median onset time of BSI would help select the appropriate prophylactic antibiotics or antifungal agents.

## Data Availability

The datasets used and/or analyzed during the current study are available from the corresponding author upon reasonable request.

## Abbreviations

AKI: Acute kidney injury
BSI: Bloodstream infections
CHF: Congestive heart failure
CIs: Confidence intervals
CoNS: Coagulase-negative staphylococcus
CRRT: Continued renal replacement therapy
EBS: Emergency bypass system
ESRD: End-stage renal disease
ECMO: Extracorporeal membrane oxygenation
HD: Hemodialysis
MDRO: Multi-drug resistant organisms
ORs: Odds ratios
PLS: Prolonged life support
VA: Veno-arterial
VAV: Veno-arterial-venous
VV: Veno-venous

## References

[1] Brodie D, Bacchetta M (2011). Extracorporeal membrane oxygenation for ARDS in adults. N. Engl. J. Med..

[2] Biffi S, Di Bella S, Scaravilli V, Peri AM, Grasselli G, Alagna L (2017). Infections during extracorporeal membrane oxygenation: Epidemiology, risk factors, pathogenesis and prevention. Int. J. Antimicrob. Agents.

[3] Della Torre V, Robba C, Pelosi P, Bilotta F (2019). Extra corporeal membrane oxygenation in the critical trauma patient. Curr. Opin. Anaesthesiol..

[4] Hsu MS, Chiu KM, Huang YT, Kao KL, Chu SH, Liao CH (2009). Risk factors for nosocomial infection during extracorporeal membrane oxygenation. J. Hosp. Infect..

[5] Kim DW, Yeo HJ, Yoon SH, Lee SE, Lee SJ, Cho WH (2016). Impact of bloodstream infections on catheter colonization during extracorporeal membrane oxygenation. J. Artif. Organs.

[6] Sun HY, Ko WJ, Tsai PR, Sun CC, Chang YY, Lee CW (2010). Infections occurring during extracorporeal membrane oxygenation use in adult patients. J. Thorac. Cardiovasc. Surg..

[7] Yu WS, Paik HC, Haam SJ, Lee CY, Nam KS, Jung HS (2016). Transition to routine use of venoarterial extracorporeal oxygenation during lung transplantation could improve early outcomes. J. Thorac. Dis..

[8] Centers for Disease Control and Prevention. Bloodstream infection event (central line-associated bloodstream infection and non-central line-associated bloodstream infection). Device-associated Module BSI; 2017.

[9] Wolfensberger A, Kuster SP, Marchesi M, Zbinden R, Hombach M (2019). The effect of varying multidrug-resistence (MDR) definitions on rates of MDR gram-negative rods. Antimicrob. Resist. Infect. Control.

[10] Bizzarro MJ, Conrad SA, Kaufman DA, Rycus P (2011). Extracorporeal Life Support Organization Task Force on Infections, Extracorporeal Membrane Oxygenation. Infections acquired during extracorporeal membrane oxygenation in neonates, children, and adults. Pediatr. Crit. Care Med..

[11] Quintana MT, Mazzeffi M, Galvagno SM, Herrera D, Boyajian GP, Hays NM (2021). A retrospective study of infection in patients requiring extracorporeal membrane oxygenation support. Ann. Thorac. Surg..

[12] Menaker J, Galvagno S, Rabinowitz R, Penchev V, Hollis A, Kon Z (2019). Epidemiology of blood stream infection in adult extracorporeal membrane oxygenation patients: A cohort study. Heart Lung.

[13] Kim GS, Lee KS, Park CK, Kang SK, Kim DW, Oh SG (2017). Nosocomial infection in adult patients undergoing veno-arterial extracorporeal membrane oxygenation. J. Korean Med. Sci..

[14] Allou N, Lo Pinto H, Persichini R, Bouchet B, Braunberger E, Lugagne N (2019). Cannula-related infection in patients supported by peripheral ECMO: Clinical and microbiological characteristics. ASAIO J..

[15] Burket JS, Bartlett RH, Vander Hyde K, Chenoweth CE (1999). Nosocomial infections in adult patients undergoing extracorporeal membrane oxygenation. Clin. Infect. Dis..

[16] Kim HS, Park S, Ko HH, Ha SO, Lee SH, Kim YK (2021). Different characteristics of bloodstream infection during venoarterial and venovenous extracorporeal membrane oxygenation in adult patients. Sci. Rep..

[17] Ko RE, Huh K, Kim DH, Na SJ, Chung CR, Cho YH (2020). Nosocomial infections in-hospital cardiac arrest patients who undergo extracorporeal cardiopulmonary resuscitation. PLoS One.

[18] Raad II, Bodey GP (1992). Infectious complications of indwelling vascular catheters. Clin. Infect. Dis..

[19] Cavayas YA, Yusuff H, Porter R (2018). Fungal infections in adult patients on extracorporeal life support. Crit. Care.

[20] De Roux Q, Botterel F, Lepeule R, Taccone FS, Langeron O, Mongardon N (2019). Candida bloodstream infection under veno-arterial ECMO therapy. Crit. Care.

[21] Vogel AM, Lew DF, Kao LS, Lally KP (2011). Defining risk for infectious complications on extracorporeal life support. J. Pediatr. Surg..

